# Measurement of ventilation and cardiac related impedance changes with electrical impedance tomography

**DOI:** 10.1186/cc9985

**Published:** 2011-01-25

**Authors:** Caroline A Grant, Trang Pham, Judith Hough, Thomas Riedel, Christian Stocker, Andreas Schibler

**Affiliations:** 1 Paediatric Critical Care Research Group, Paediatric Intensive Care Unit, Mater Children's Hospital, 550 Stanley Street, South Brisbane, Queensland 4101, Australia; 2 Institute of Health and Biomedical Innovation, Queensland University of Technology, 96/110 Victoria Park Road, Kelvin Grove, Queensland 4059, Australia; 3 Paediatric and Neonatal Intensive Care, Department of Paediatrics, Inselspital, University Children's Hospital,University of Bern, CH-3010 Bern, Switzerland

## Abstract

**Introduction:**

Electrical impedance tomography (EIT) has been shown to be able to distinguish both ventilation and perfusion. With adequate filtering the regional distributions of both ventilation and perfusion and their relationships could be analysed. Several methods of separation have been suggested previously, including breath holding, electrocardiograph (ECG) gating and frequency filtering. Many of these methods require interventions inappropriate in a clinical setting. This study therefore aims to extend a previously reported frequency filtering technique to a spontaneously breathing cohort and assess the regional distributions of ventilation and perfusion and their relationship.

**Methods:**

Ten healthy adults were measured during a breath hold and while spontaneously breathing in supine, prone, left and right lateral positions. EIT data were analysed with and without filtering at the respiratory and heart rate. Profiles of ventilation, perfusion and ventilation/perfusion related impedance change were generated and regions of ventilation and pulmonary perfusion were identified and compared.

**Results:**

Analysis of the filtration technique demonstrated its ability to separate the ventilation and cardiac related impedance signals without negative impact. It was, therefore, deemed suitable for use in this spontaneously breathing cohort.

Regional distributions of ventilation, perfusion and the combined ΔZ_V_/ΔZ_Q _were calculated along the gravity axis and anatomically in each position. Along the gravity axis, gravity dependence was seen only in the lateral positions in ventilation distribution, with the dependent lung being better ventilated regardless of position. This gravity dependence was not seen in perfusion.

When looking anatomically, differences were only apparent in the lateral positions. The lateral position ventilation distributions showed a difference in the left lung, with the right lung maintaining a similar distribution in both lateral positions. This is likely caused by more pronounced anatomical changes in the left lung when changing positions.

**Conclusions:**

The modified filtration technique was demonstrated to be effective in separating the ventilation and perfusion signals in spontaneously breathing subjects. Gravity dependence was seen only in ventilation distribution in the left lung in lateral positions, suggesting gravity based shifts in anatomical structures. Gravity dependence was not seen in any perfusion distributions.

## Introduction

Electrical Impedance Tomography (EIT) is an emerging technique for bed-side assessment of ventilation distribution. It has been shown to be able to distinguish regional distributions of both ventilation and perfusion [1,2]. Several methods have been suggested to separate these signals, the simplest being breath holding to remove respiratory changes [3], which also removes the ability to assess cardio-pulmonary interaction. Alternatively ECG gating and frequency filtering has been suggested, which would allow acquisition of the perfusion components of the EIT signal without respiratory interference [4-6].

Recently, Frerichs *et al. *examined the distribution of lung perfusion in mechanically ventilated adults during bilateral and unilateral ventilation of the left and right lungs [2]. They utilised a band pass filtering technique and linear regression fit to establish functional regions of interest (ROI), identifying two regions - the left and right lung. This method appears sound in identifying functional areas of lung tissue; however, subjects were mechanically ventilated and the breath rate manipulated so as not to interfere with the frequency characteristics of the heart rate. While this may be feasible in some mechanically ventilated subjects, on the whole it is not practical clinically. It, therefore, remains to be seen whether this method can be extended to a spontaneously breathing cohort.

Fagerberg *et al. *also examined perfusion using EIT and calculated a V/Q ratio on anaesthetised piglets [1,7]. While highlighting the problems with differentiating ventilation and perfusion signals in EIT, they proposed instead to circumvent the issue by recording perfusion during a short apnoea. The breath-hold approach captures the cardiac related impedance signal without the need for filtering, but lacks the ability to measure the interactions between ventilation and cardiac signals. While interesting, again this is not exactly practical in a clinical setting.

In this study, therefore, it is aimed to extend Frerichs functional filtration method to spontaneously breathing adults and assess the regional distributions of ventilation and perfusion. By incorporating a breath hold period, similar to Fagerberg's apnoea, cardiac related impedance changes can be easily identified and the impact of filtering on ventilation/perfusion relationships better analysed. This study presents a stepwise approach, extending previously suggested filtering techniques with new methods to assess ventilation/perfusion relationships using EIT.

## Materials and methods

Ten healthy adults (21 to 52 years) were recruited from the staff of the Paediatric Intensive Care Unit at the Mater Children's Hospital, South Brisbane, Australia. The study was approved by the Human Ethics Committee of the Mater Health Services and participant consent was obtained.

The participants were to breathe normally for 30 seconds followed by breath holding for 30 seconds while in a supine position. ECG data were recorded simultaneously for these measurements. EIT data were also recorded for a period of 10 minutes of spontaneous breathing in supine, prone, left- and right-lateral positions, from which a period of steady breathing (5 to 10 breaths) was used for analysis.

A Göttingen GoeMF II EIT tomograph (CareFusion, San Diego, CA, USA) was used with a frame rate of 44 Hertz (Hz). EIT methodology has been extensively described elsewhere [8-10]. EIT measures regional impedance change using small current injections, 16 electrodes were placed around the chest at nipple level. Dedicated software was used for data acquisition and reconstruction of EIT images (MATLAB^® ^7.7.0, The Mathworks, Inc., Natick, MA, USA).

### Analysis of filtering technique on cardiac related impedance signal

**Figure 1:**
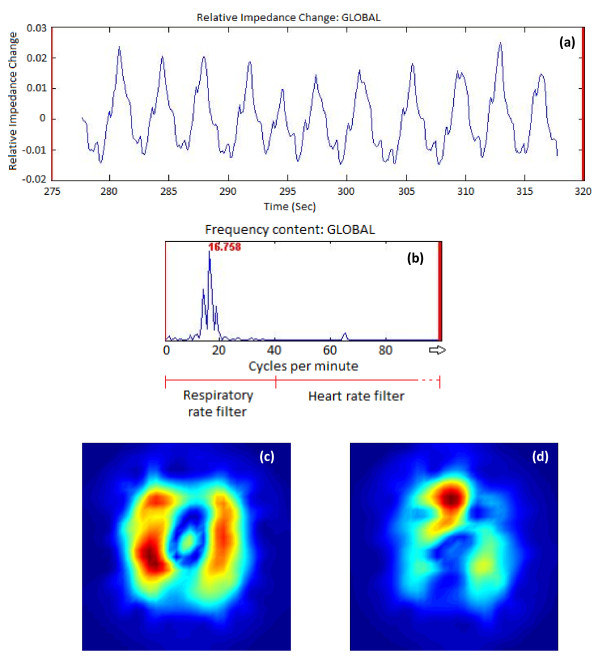
**Filtering of the EIT signal**. **(a) **The original time course of impedance change of a subject during spontaneous breathing with no filtering applied. **(b) **The Fast Fourier Transform (FFT) power spectrum of this signal showing the frequency characteristics. The peak frequency highlighted is the respiratory rate, band pass filtering for the respiratory rate was set from 2/minute to 2.5 times the respiratory rate - in this case 42/minute. The heart rate filtered data were extracted using a band pass filter above this rate, that is, 42 to 400/minute. **(c) **The standard deviation image generated when filtering around the respiratory rate. **(d) **The standard deviation image generated when filtering around the heart rate.

A slightly modified version of Frerichs *et al.*'s [2,11] filtration technique was used to separate respiratory and perfusion related impedance changes of the EIT signal. First, regions within the EIT image identifiable as functional lung (ROI_Lung_) were established. During spontaneous breathing a Fast Fourier Transformation (previously described [12]), was performed and a band pass frequency filter applied to include the subject's respiratory peak frequency and its second harmonic (Figure 1). The lower limit was set at two breaths/minute and the upper limit at 2.5 times the respiratory rate. ROI_Lung _was then defined as any region in which the impedance signal was greater than 20% of the peak impedance signal [13].

The regions of functional lung tissue described by ROI_Lung _were then outlined on the raw image during the breath hold (unfiltered). A region of high impedance change outside the ROI_Lung _was identified as ROI_Heart_.

Two measures of the coherence of two signals are the slope of the linear regression fit between them (slope) and the phase angle (α). When a linear regression fit is performed between two signals the slope of the line created will be either positive (in phase behaviour) or negative (out of phase). The phase angle then describes the temporal synchronicity of the two signals, and gives an α in degrees (ranging from 0 to 360°) describing this difference. Phase angles in the range of 90 to 270° are broadly regarded as being out of phase.

The established ROI_Lung _and ROI_Heart _signals were analysed for slope and α under three circumstances: i) During breath hold, unfiltered; ii) During breath hold, band pass filtered to exclude respiratory signal and include the perfusion signal ("HR filter" approximately 40 to 400/minute); iii) During spontaneous breathing, HR filter (as in ii, approximately 40 to 400/minute).

The slope and α were calculated in each of these cases across the four quarters of the image (anterior-left, -right, posterior-left, -right) and are shown in Table 1.

**Table 1:** Phase angle α and slopes for perfused lung quadrants in comparison to ROI_Heart _while filtered around the heart rate.

		Phase angle α (degrees)				Slope of linear regression fit			
		Ant-R	Ant-L	Post-R	Post-L	Ant-R	Ant-L	Post-R	Post-L
Breath hold period unfiltered	Mean	181	152	180	153	-0.75	-0.53	-0.98	-0.44
	CI	40	55	41	54	0.58	0.23	0.98	0.31
Breath hold period filtered	Mean	159	152	159	157	-0.53	-0.45	-0.58	-0.36
	CI	11	13	11	10	0.15	0.20	0.16	0.15
Spontaneous breathing filtered	Mean	167	159	172	168	-0.50	-0.49	-0.50	-0.37
	CI	7	11	8	7	0.09	0.16	0.10	0.12

All lung quadrants had phase angles close to 180 degrees and negative slopes indicating reversed ΔZ behaviour. Neither filtering of the impedance signal nor respiration impacted on the slopes (*P *= ns, two-way-ANOVA). Ant L/R, anterior left/right; CI, confidence interval; Post L/R, posterior left/right.

The synchronicity of the band pass filtered signal in ii and iii, with the recorded ECG signal was also examined.

### Comparison of body position on ventilation and perfusion distribution

With a region of functional lung determined (ROI_Lung_) the application of various band pass filters was then used to separate out the respiratory and perfusion related impedance changes.

As used previously, a band pass filter surrounding the respiratory rate (2/minute-2.5xRR) was used to extract the respiratory impedance changes (ΔZ_V_), and a band pass filter surrounding the heart rate, (HR filter) (approximately 40 to 400/minute) was used to extract the perfusion related impedance changes (ΔZ_Q_).

These filters were applied to a period of steady breathing (5 to 10 breaths) in each position (supine, prone, left and right lateral).

Using these data, analyses were carried out on the respiratory (ΔZ_V_) and perfusion (ΔZ_Q_) signals separately and combined into a ΔZ_V_\ΔZ_Q _ratio on a pixel by pixel basis. To calculate a ΔZ_V_\ΔZ_Q _the data were first normalised (the ΔZ_Q _signal is several magnitudes smaller than the ΔZ_V _signal). An image of the regional ΔZ_V_/ΔZ_Q _was generated by dividing the normalised ventilation value by the normalised perfusion value for each pixel. In this way the ΔZ_V_\ΔZ_Q _is not like a traditional VQ ratio but rather is a ratio of maximal ventilation to maximal perfusion, with a value of 1 occurring in a region in which the proportion of ventilation and perfusion are matched, that is, ΔZ_Vmax_/ΔZ_Qmax _OR ΔZ_Vmin_/ΔZ_Qmin_.

The sum of the pixel values of ΔZ_V_, ΔZ_Q _and ΔZ_V_\ΔZ_Q _was calculated for dependent and non-dependent lung regions (each comprising half the image) in each position. Profiles of ΔZ_V_, ΔZ_Q_, and ΔZ_V_\ΔZ_Q _from right to left and posterior to anterior in 32 slices were also determined in each position [14,15].

### Statistics

All results are presented as mean with confidence interval (CI). A two-way ANOVA was used to compare the slopes and phase angles of the impedance signal; during ventilation vs. breath-hold and for filtered vs. non-filtered. A one-way ANOVA was used to compare regional differences for ventilation and cardiac related impedance changes, both from dependent to non-dependent regions within positions, and between positions.

## Results

### Filtration technique

Examination of the slopes and α's calculated across the lung during the breath hold with/without filtering and during breathing with filtering allowed the effects of the filtering technique on the perfusion signal to be quantified. This analysis showed no significant effect on the perfusion signal from either the filtering process or the presence of the respiratory signal (*P *= ns, two-way-ANOVA). As seen in Table 1 all ROI_Lung _regions showed inverse impedance behaviour to ROI_Heart _with negative slopes and α between 152° and 181°.

### Regional distribution of ventilation and perfusion

**Figure 2:**
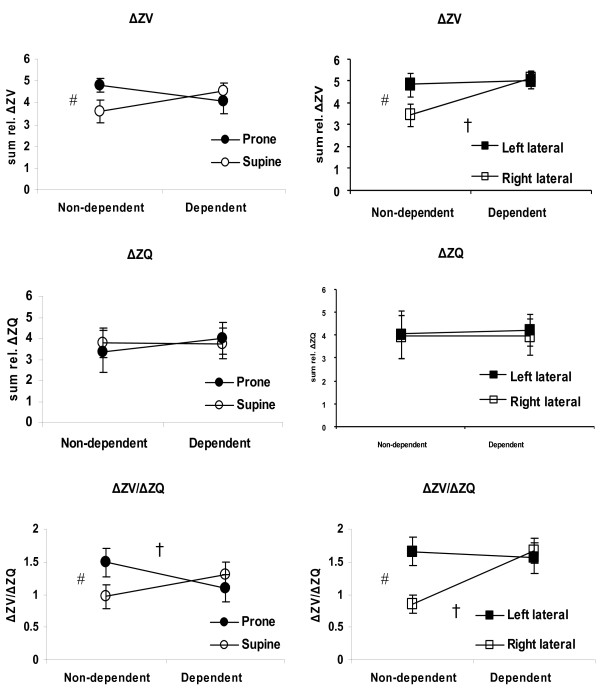
**Sum of relative impedance change in dependent and non-dependent lung regions**. The sum of ΔZ_Q _and ΔZ_V _and ΔZ_V_/ΔZ_Q _in dependent and non-dependent regions for supine, prone, left and right lateral position (mean and confidence interval (CI)). ^# ^indicates a significant difference between positions in the non-dependent lung and ^† ^indicates significant difference within the same position between dependent and non-dependent lung (*P *< 0.05).

Figure 2 shows the sum of ΔZ_V_, ΔZ_Q _and the calculated ΔZ_V_/ΔZ_Q _for the dependent and non-dependent lung in all positions. Comparison within each position showed significant differences (*P *< 0.05) between the dependent and non-dependent lung in ventilation distribution (right lateral position) and in ΔZ_V_/ΔZ_Q _(prone and right lateral positions).

Comparison between positions showed significant differences in the non-dependent lung in ventilation and ΔZ_V_/ΔZ_Q_. In both cases prone and left lateral positions were significantly higher (than supine and right lateral respectively). The ΔZ_Q _distribution was not significantly influenced by position.

**Figure 3:**
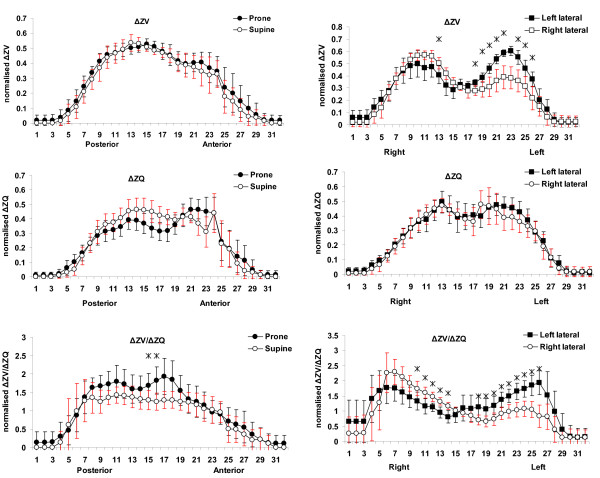
**Profiles of normalised impedance change along the gravity axis**. Profiles of the sum of normalised impedance change across the lung. The horizontal axis of each plot shows the slice or pixel row/column number from posterior to anterior or right to left in each position. The upper two plots show the distribution of ΔZ_V_, the central two the distribution of ΔZ_Q _and the lower two the distribution of ΔZ_V_\ΔZ_Q_. A significant difference between the two positions within a region is indicated with a *.

Figure 3 shows profiles of normalised ΔZ_V_, ΔZ_Q _and ΔZ_V_/ΔZ_Q _in each position. Significant differences were seen between positions - in ΔZ_V _distribution (lateral positions) and in ΔZ_V_/ΔZ_Q _(lateral positions and prone/supine). Significantly greater ventilation can be seen in the left lung in the left lateral position.

The effect of these ΔZ_V _differences on the ΔZ_V_/ΔZ_Q _can also be seen with significant differences in both the left and right regions of the chest with greater values seen in the dependent region.

In prone and supine positions the ΔZ_V_/ΔZ_Q _is higher in the posterior regions of the lung. Prone position results in higher values than supine across most of the posterior slices, though the difference is only significant in two of the more central slices.

Very little change was seen in the ΔZ_Q _profiles, with those for the lateral positions being remarkably similar.

## Discussion

Previous studies suggested either a breath-hold, or a signal filtering approach for separating the two sources of impedance change [3]. The breath-hold approach captures the cardiac related impedance signal without the need for filtering, but lacks the ability to measure the interactions between ventilation and cardiac signals. The filtering approach is flawed by neglecting important information on heart beat variability, and on cross-talk between ventilation and heart rate signals by a potential direct overlap of harmonics but allows the inclusion of phase information.

In this study, ventilation and perfusion data were successfully separated out of the combined EIT signal and analysed. The filtration technique used built on methods described by Frerichs *et al. *and extended the technique into a spontaneously breathing population in which higher harmonics of ventilation would likely overlap and swamp the cardiac signal [2]. It was shown that there was no significant difference to the perfusion signal introduced by the filtering technique during a breath hold, or when filtering out a ventilation signal. Making the technique suitable for use on the spontaneously breathing cohort as well as on patients in which the ventilation rate cannot be adjusted or an apnoea induced for the sake of gathering data.

### The validity of the cardiac related impedance signal

EIT measures regional changes in air volume and distribution in the lung, for example, ventilation, with high accuracy, but less is known of its capacity to measure perfusion [2]. In a porcine model Fagerberg *et al. *measured stroke volume with a pulmonary artery catheter and compared it to pulse-synchronous impedance changes measured with EIT [1]. The beat-to-beat pulmonary perfusion was accurately measured with EIT over a large range of stroke volumes.

**Figure 4:**
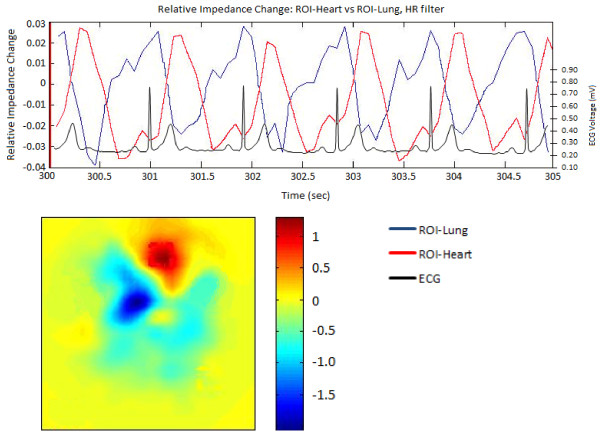
**Heart rate filtered data with ECG trace**. Filling Capacity Image and superimposed relative impedance change trace taken while filtered at the heart rate range. The heart (ROI-Heart) is seen in red at the top of the filling capacity image and its time course is traced in red. The blue regions and time course are that of the perfused lung (ROI-Lung). The simultaneously sampled ECG trace is shown on top of the impedance time course for comparison.

Visual analysis of the ROI_Lung _showed perfect alignment of the cardiac related impedance changes with the ECG. A significant phase lag between the ROI_Heart _and each ROI_Lung _could be seen, thus demonstrating the time course of blood moving away from the heart (Figure 4, Table 1).

It is uncertain as to what effect the cardiac structures have on the impedance signal [6]. It is possible that mechanical interaction of the heart with the surrounding lung tissue is responsible for the changes in impedance, rather than the pulsatile intrapulmonary blood volume. Assuming that the pulsatile impedance signal within the lung is caused by mechanical interaction only, then an increase in the impedance signal would be expected during systole as the lung expands while the heart contracts. Our study showed the opposite. During heart contraction the impedance of ROI_Heart _increased as a result of reduced blood volume, that is, decreased conductivity, while simultaneously the impedance value in the lung decreased as a result of the increased blood volume in the lung, that is, increased conductivity. The calculated slopes of ROI_Lung _were negative demonstrating that impedance changes were caused by pulsatile blood volume. The calculated phase angles showed a significant phase lag between ROI_Heart _and ROI_Lung_, which supports the motion that the pulsatile impedance changes may represent perfusion.

The same phase relationship between ROI_Heart _and ROI_Lung _during breathing and breath-hold was found. Hence, we demonstrated that filtering did not impact on the phase shift of the cardiac related impedance signal within the lungs (Table 1).

### Ventilation distribution

Previous studies have shown ventilation distributed preferentially towards the dependent lung and attributed this to gravity [18]. While this may be the case in upright positions it remains to be seen if gravity still plays a role when horizontal.

The profiles of ΔZ_V _shown in Figure 3 in fact show a lack of gravity dependence in supine/prone positions, with the two profiles being virtually identical. The profiles from the lateral positions do, however, show a difference, with greater ventilation in the left lung in left lateral position, though only a slight change in the distribution to the right lung rather than the complete shift gravity dependence might imply.

If gravity had an effect on the air flow itself these findings would make no sense, reversing patterns would be seen between positions. Instead it can be inferred from these plots that gravity plays a role in ventilation distribution across the chest through its effect on anatomy.

Anatomically there is very little change in the chest from prone to supine positions, as evidenced by the similarity in the profiles. When changing lateral positions however large changes in anatomy occur with the shift of gravity direction. As the heart is already in the left side of the chest its impact on ventilation in left lateral position is minimised. Ventilation distribution is compromised in right lateral position however as gravity causes a shift in the position of mediastinal organs.

#### Perfusion distribution

If gravity plays a role in blood volume, regions of the lung at the same height (iso-heights) should have similar blood volume. Similar to ΔZ_V_, gravity had little effect on ΔZ_Q _distribution, and the profiles showed no significant regional differences (Figure 3). This agrees with a previous study using injected microspheres in dogs, showing considerable blood volume heterogeneities within iso-height planes [16].

#### ΔZ_V_/ΔZ_Q _distribution

Unlike traditional VQ which relates ventilation and perfusion rates in L/minute the ΔZ_V_/ΔZ_Q _compares the amplitude of impedance change after normalisation of the two signals. A ΔZ_V_/ΔZ_Q _of 1 does not imply that the components have the same magnitude change, but rather that the proportion of ventilation and perfusion are matched, that is, ΔZ_Vmax_/ΔZ_Qmax _OR ΔZ_Vmin_/ΔZ_Qmin_. Although only relative changes can be detected, this approach allows investigation of the impact of gravitational factors on ventilation and cardiac related ΔZ.

As would be expected from the ventilation and perfusion distributions there is little difference between supine and prone positions. Across the central to posterior portions of the lung supine position in particular has a very consistent relationship of around 1.2 to 1.4. The values in prone position tend to be higher (1.5 to 1.9) across this portion of the chest though the differences are generally not significant; significance is only reached in two of the central regions as a by-product of a non-significant drop in perfusion in these regions.

The distribution across the chest in lateral positions is however quite different. Significant differences can be seen between left and right lateral positions in both the left and right regions of the chest. This is to be expected because of the gravity dependant changes in ventilation, and lack of gravity dependence in perfusion distribution. Had the perfusion distribution shown a similar pattern of gravity dependence to the ventilation distribution the ΔZ_V_/ΔZ_Q _would have been more consistent across the lung as is seen in supine and prone positions (where neither ventilation nor perfusion show gravitational effects).

Instead the ΔZ_V_/ΔZ_Q _pattern follows the ventilation distribution pattern with each position significantly higher in its dependent lung (that is, left lung in left lateral). Again the values across the central regions of the lung tend to be high (up to 2.3) in the dependent lung in each case.

As a value of 1 in this ΔZ_V_/ΔZ_Q _calculation is a matching of comparative amplitude the high values seen across the lung in all positions suggests a greater or broader distribution of ventilation than perfusion across the lung, that is, more pixels in the higher ranges of ventilation than perfusion. This suggests that perhaps a simple normalisation of the signals is not the most appropriate technique for making the two signals comparative, but that further parameters such as the standard deviation of the values also may need to be considered.

#### Limitations

The measurement of ventilation and perfusion with EIT will remain a complex task. The interaction of these two physiological events will impact on the accuracy of impedance measurements, which are only surrogates for true ventilation and perfusion. ΔZ_V_/ΔZ_Q _of different lung regions were assessed by normalising the impedance signals of respiration and lung perfusion. A ΔZ_V_/ΔZ_Q _of 1 does not imply that both components of the relationship have the same flow rate but that they share the same quantitative relationship to the maximal amplitude of measured impedance in the specific frequency range.

Gravity dependent changes in ΔZ_V_/ΔZ_Q _could be demonstrated (particularly in lateral positions), similar to those found using other measurement techniques with greater spatial resolution such as electron-beam CT [17] or radio-labelled tracers [18]. It is acknowledged that no direct reference method has been used to compare the lung perfusion signal, but the use of any other imaging technique with x-rays or radio-labelled tracers has been denied by our ethical standards. Other filtering techniques using dynamic frequency filtering could further improve the separation of the ventilation and perfusion signals and therefore improve the ΔZ_V_/ΔZ_Q _[19]. Precise regional assessment of ventilation and cardiac related impedance changes are further complicated by the low resolution and interregional blurring effect of EIT. The proposed ROI definition of our study will not identify atelectatic regions as lung tissue and these areas cannot be analysed.

The use of the term 'perfusion' for this heart rate synchronous impedance signal is an area of some contention. Frerichs *et al. *[3] have also described this signal as perfusion and present further data supporting this terminology. It is, however, acknowledged that there may be other factors involved such as the mechanical transmission of pressure waves onto the surrounding tissue from the heart beating. The impedance signal generated by this mechanical interaction, however, would have a distribution which diminishes with distance from the heart, much like a stone in a pond causing ripples. This is not the pattern of impedance distribution that is seen at this frequency range.

## Conclusions

In this study we examined previously used filtration techniques and extended and adapted them to a spontaneously breathing healthy adult cohort. Examination of the effects of the filtration process determined that the method described was suitable for filtering and separating regional ventilation and perfusion related impedance changes.

The regional distributions of ΔZ_V_, ΔZ_Q _and ΔZ_V_/ΔZ_Q _were examined in supine, prone, left- and right-lateral positions, and the effects of gravity determined. Significant gravity dependence was not seen in any position. Gravity dependence was only seen in ΔZ_V _in lateral positions, likely caused by the shift in mediastinal structures. ΔZ_V_/ΔZ_Q _distributions were above one for non-peripheral regions of the lung in all positions. In supine and prone position the ΔZ_V_/ΔZ_Q _was quite consistent across the lung regions whereas the lateral positions showed significantly higher values in the respective dependent regions.

## Key messages

• It is possible to distinguish between lung ventilation and perfusion using Electrical Impedance Tomography (EIT).

• A modified filtration technique can effectively separate respiratory and perfusion related impedance changes of the EIT signal in spontaneously breathing subjects.

• Gravity dependence was not seen in any perfusion distributions in spontaneously breathing adults.

## Abbreviations

ANOVA: analysis of variance; CI: confidence interval; CT: computed tomography; ECG: electrocardio graph; EIT: electrical impedance tomography; HR: heart rate; HZ: hertz; ROI: region of interest (lung or heart); ΔZ: impedance change; ΔZ_V_/ΔZ_Q_: ventilation impedance change divided by cardiac impedance change.

## Competing interests

The authors declare that they have no competing interests.

## Authors' contributions

CG assisted with study design, data processing, analysis and interpretation, and drafting the manuscript. TP assisted with data collection, software engineering, and data processing. JH assisted with participant recruitment, data collection, data interpretation, and drafting the manuscript. CS assisted with study design and data interpretation. TR and AS assisted with study design, data interpretation, and drafting the manuscript. All authors read and approved the final manuscript.
